# Joint extraction of wheat germplasm information entity relationship based on deep character and word fusion

**DOI:** 10.1038/s41598-024-59796-9

**Published:** 2024-05-06

**Authors:** Xiaoxiao Jia, Guang Zheng, Chenyang Dong, Shiyu Xi, Lei Shi, Shufeng Xiong, Xinming Ma, Lei Xi

**Affiliations:** 1 College of Information and Management Sciences, Henan Agriculture University, Zhengzhou, 450046 China; 2 Henan Engineering Laboratory of Farmland Environmental Monitoring and Control, Zhengzhou, 450046 China; 3 University of London, London, WC1E 7HU UK

**Keywords:** Wheat germplasm information, Character and word fusion, Entity relation extraction, Joint extraction, Cascading pointer network, Data mining, Data processing, Machine learning, Information technology

## Abstract

The verified text data of wheat varieties is an important component of wheat germplasm information. To automatically obtain a structured description of the phenotypic and genetic characteristics of wheat varieties, the aim at solve the issues of fuzzy entity boundaries and overlapping relationships in unstructured wheat variety approval data, WGIE-DCWF (joint extraction model of wheat germplasm information entity relationship based on deep character and word fusion) was proposed. The encoding layer of the model deeply fused word semantic information and character information using the Transformer encoder of BERT. This allowed for the cascading fusion of contextual semantic feature information to achieve rich character vector representation and improve the recognition ability of entity features. The triple extraction layer of the model established a cascading pointer network, extracted the head entity, extracted the tail entity according to the relationship category, and decoded the output triplet. This approach improved the model’s capability to extract overlapping relationships. The experimental results demonstrated that the WGIE-DCWF model performed exceptionally well on both the WGD (wheat germplasm dataset) and the public dataset DuIE. The WGIE-DCWF model not only achieved high performance on the evaluation datasets but also demonstrated good generalization. This provided valuable technical support for the construction of a wheat germplasm information knowledge base and is of great significance for wheat breeding, genetic research, cultivation management, and agricultural production.

## Introduction

Wheat germplasm resources play a vital role in developing new wheat varieties and improving quality. It also provides a solid foundation for food production and food safety^1^. Wheat variety validation information provides valuable data on various aspects, such as agronomic characteristics, morphological traits, resistances, and genetic relationships. This information is an essential component of wheat germplasm information and holds significant reference value for breeding improvement, cultivation management, and the utilization of excellent varieties. However, managing, storing, retrieving, publishing, and utilizing the abundance of unstructured text data in wheat variety validation information has become increasingly challenging and complex. Joint entity and relation extraction is a critical subtask in information extraction that aims to extract entities and their relationships from unstructured text data^2,3^. This process transforms unstructured text into structured knowledge in the form of "(Entity1, Relationship, Entity2)". By capturing the interactions between entities and relationships, joint entity and relation extraction can help address issues such as error propagation in pipeline extraction^4,5^. It provides crucial support for knowledge management, retrieval, and applications. While existing joint entity and relation extraction methods have achieved promising results, the wheat germplasm information domain presents specific challenges. In the wheat germplasm information domain, fuzzy entity boundary positioning is a challenge due to the presence of specialized domain entities and special characters, including units. Although the introduction of word information has aided in segmenting entity boundaries and improving entity recognition^6,7^, it has only scratched the surface in terms of integrating word information and has not fully harnessed the representational power of BERT to fuse word semantic information effectively. Additionally, in the domain of wheat germplasm information, the relationships between entities in textual data are often intertwined, resulting in a large number of overlapping triples. While the cascading pointer network proposed^8^ effectively addresses the issue of overlapping relationships, it still struggles to capture semantic information adequately.

To tackle the challenges related to fuzzy entity boundaries and overlapping relationships in the domain of wheat germplasm information effectively, this research introduced a novel approach known as WGIE-DCWF. Firstly, to address the limited availability of wheat germplasm information datasets, a fine-grained wheat germplasm dataset was created. This dataset served as a valuable resource for training and evaluating the proposed WGIE-DCWF model, thereby enhancing the available data resources in this domain. Secondly, at the encoding layer, the model incorporated a deep character and word fusion module alongside a bidirectional semantic feature fusion module. These modules synergistically enriched the character vector information, enabling more accurate localization of entity boundaries within the complex and specialized wheat germplasm information domain. By effectively integrating the intricate interplay between characters, words, and bidirectional semantic features, the model significantly enhanced entity boundary detection. Finally, to effectively handle the challenge of overlapping relationships, a cascading pointer network was employed. This network sequentially extracted relationships between entities, successfully addressing the issue of overlapping triplets, and provided more precise and reliable relation extraction. The proposed WGIE-DCWF model exhibited exceptional performance on both the WGD and the widely recognized public dataset DuIE. The experimental results demonstrated the superiority and effectiveness of the proposed approach in the joint entity and relation extraction task within the wheat germplasm information domain. In summary, the main contributions of this research could be summarized as follows:

1. The WGD was constructed using a three-round annotation method to manually create 23 types of entities and 25 types of relationships. This dataset helped alleviate the scarcity of data in the wheat germplasm information domain.
2. We propose the WGIE-DCWF model, which achieves a deep fusion of characters and words using the Transformer encoder of BERT. Additionally, it employs BiLSTM for bidirectional extraction of contextual semantics, enhancing entity recognition capability. Furthermore, a cascading pointer network is established, elevating the model’s ability to extract overlapping triplets.
3. The WGIE-DCWF model was tested on both the WGD and the DuIE datasets. It demonstrated good performance on both datasets and achieved F1 scores of 93.59% and 77.73%, respectively. These results validated that the model improved the extraction of entity relationships in wheat germplasm information data and exhibited good generalization capabilities.

## Related works

Joint entity and relation extraction methods played a crucial role in modeling textual information, enabling automated identification of entities, entity types, and specific relationship categories between entities. These methods provided valuable technical support for downstream tasks such as knowledge graph construction, intelligent question answering, and semantic search^4^. Currently, mainstream deep learning-based entity and relation extraction methods could be categorized into pipeline extraction and joint extraction, based on the order of entity recognition and relationship extraction tasks. While pipeline extraction methods^9,10^ were simple and flexible, they were prone to error propagation and entity redundancy issues. As a result, researchers have increasingly focused on joint extraction methods.

Zheng^11^ initially put out a technique for converting joint extraction into sequence labeling, but the overlapping triples could not be extracted because of the labels’ nearest matching. The VOE designation was proposed by Tang^12^. To resolve the issue of overlapping relationships, the entity labeled with the label can match with other entities numerous times. However, the complicated labeling methods make joint extraction more challenging. To address the issue of overlapping relationships, Zeng^13^ introduced the CopyRE model, which duplicates entities multiple times using a replication mechanism. However, the complex decoding structure of the model resulted in poor performance on local information. Xu and Gao^14,15^ proposed embedding relationships to directly obtain head and tail entities. However, identifying candidate relationship categories remains a challenging task. To address this issue, Wei^8^ introduced the CASREL method, which treats the relationship as a mapping from the head entity to the tail entity, effectively resolving the problem of relationship overlap. However, the model’s extraction of semantic information is limited, as it solely relies on BERT for encoding. In response to this limitation, Shen^16^ enhanced the CASREL by incorporating an attention layer, enabling the extraction of multiple relationships. Hu^17^ further improved the approach by introducing the CLN network layer, which strengthened the extraction of head entities and their corresponding relationship-tail entities. Yu^18^ incorporated both positive and negative relationships to enhance triplet extraction. Wang^19^ leveraged entity-type information to tackle the challenges of nested entities and relationship overlap. While these methods effectively addressed the problem of relationship overlap, they mainly focused on character-level semantic information and did not fully integrate character and word information.

In the realm of text vector representation, character vectors fall short in conveying intricate semantic information. On the other hand, word vectors encapsulate both the boundary and semantic details of words. Hence, a fusion of character and word vectors proves advantageous in tackling the challenge of ambiguous entity boundaries. Zhang^20^ utilized Lattice LSTM to embed word information into character representations, effectively mitigating word segmentation errors. However, it should be noted that Lattice LSTM was limited to LSTM networks and had relatively lower computational performance. Based on Transformer, Li^21^ combined word data to parallelize the model and accelerate execution performance. To tackle the challenge of relationship extraction more effectively, Ge^6^ combined word information and BERT character information for the joint entity and relation extraction task. Word and character information were combined in the before-mentioned task. Although it enhanced entity recognition, it merely integrated word and character information at the model level. It did not fully leverage the potential of BERT to exploit the rich word semantic and character information. In this study, we integrated word information within the BERT, enabling a deep interaction between word information and character information through BERT’s multi-layer Transformer encoder structure. This enhances the details of character encoding features within BERT.

In recent years, the application of joint entity and relation extraction techniques in the agricultural domain has significantly contributed to the advancement of agricultural informatization. Zhang^22^ introduced rules amendment, which improved the performance of wheat pest and disease named entity recognition. Li^23^ employed multi-source information fusion to enhance character vectors but only achieved preliminary named entity recognition in the field of crop diseases and pests. Wu^24^ adopted a pipeline approach, utilizing BiLSTM-CRF for named entity recognition and CNN for relation extraction. It successfully extracted connections between rare plants and animals in China, including their distribution areas, endangered levels, and scientific classifications. Nonetheless, this approach suffered from issues related to entity redundancy, and the relation extraction model was relatively simplistic. To address the problem of relation overlap in the domain of wheat diseases and pests, Wu^25^ proposed the concept of a main entity. However, the labeling scheme employed in this approach was complex, which could potentially limit its practical applicability. Zhou^26^ made improvements to the CASREL method and enhanced the performance of the model on a self-built dataset of rice fertilization by using unit annotators and hidden layers. This effectively resolved the issue of overlapping triplets in the domain of rice fertilization.

This paper addressed the issues of entity boundary ambiguity and relation overlap in wheat germplasm information extraction. To tackle these challenges, the paper introduced word information and proposes deep character and word fusion. It further utilized a cascading pointer network to construct the WGIE-DCWF model. The goal was to automatically extract phenotypic and genetic descriptions of wheat varieties, providing technical support for the construction of a wheat germplasm information knowledge base. Additionally, the model aimed to provide data support for wheat germplasm pedigree analysis and variety recommendation.

## Wheat germplasm dataset

To address the issue of data scarcity in the field of wheat germplasm information, this study utilized wheat variety verification information provided by credible research institutions such as the China Seed Industry Big Data platform. By employing ontology modeling and data annotation, the researchers constructed a wheat germplasm dataset called WGD, which contains 3000 wheat varieties and 11,681 data entries.

### Ontology-based modeling of wheat germplasm information

**Figure 1:**
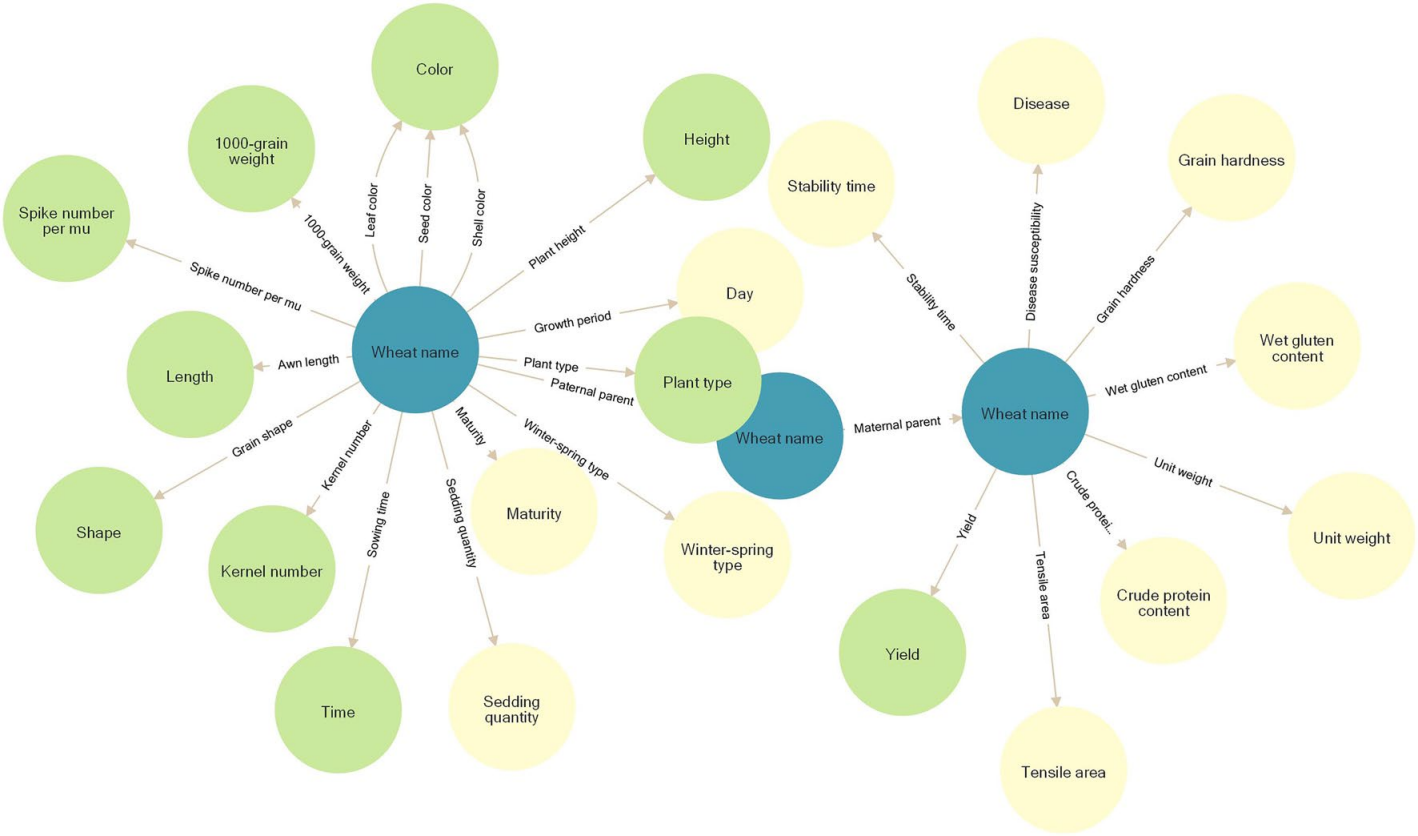
Ontology of wheat germplasm information. Nodes represent entity types, and edges represent relationship types.

This paper combines expert guidance from the wheat germplasm information field and the work of Javris^27^ to construct an ontology for the wheat germplasm information domain. The ontology effectively captures the essential characteristics of agronomic traits, morphological traits, resistance, and kinship information found in wheat germplasm data. It had 25 types of relationships and 23 types of entities, including kernel number, winter-spring type, plant height, and wheat name. The terms "paternity" and "maternal" were used to describe the relative between the various wheat types. Fig. 1 displayed the wheat germplasm information ontology.

### The annotation strategy for wheat germplasm information data

To obtain a high-quality WGD, this paper adopted the pointer network annotation method. Based on the doccano online annotation platform (https://github.com/doccano), three iterations of manual annotation and error correction were performed to ensure the accuracy of the annotated data.

**Figure 2:**
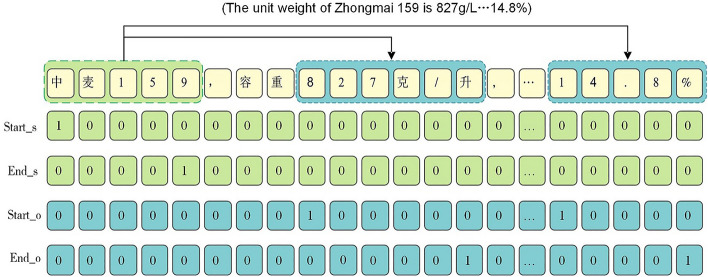
"01" labeling scheme diagram with separated head and tail entities. Label the input sentence with the start and end positions of the head entity "Zhongmai 159" and the tail entities "827g/L" and "14.8%". In the labeling sequence, mark 1 if it is the start or end position of an entity, otherwise mark 0.

The annotation strategies for entity relation extraction included sequence labeling and pointer network annotation. In sequence labeling, the principle of proximity was used to annotate entity relations. This meant that when an entity had multiple relations with other entities in the context, the relation was assigned to the nearest entity, making it difficult to effectively handle relation overlap. On the other hand, the pointer network annotation method used a "01" labeling scheme, where the starting and ending tokens of an entity were labeled as "1", and the remaining tokens were labeled as "0". The entity was then output by concatenating the starting and ending tokens. As shown in Fig. 2, the corpus contained two triples with overlapping relations. In the annotation, the head entity "Zhongmai 159" and the tail entities "827 g/L" and "14.8%" were labeled separately, with the starting and ending positions marked as "1" and other positions marked as "0". The pointer network annotation method could effectively solve the problem of relation overlap and require fewer labels, thus reducing the complexity during prediction.

## Model architecture construction

**Figure 3:**
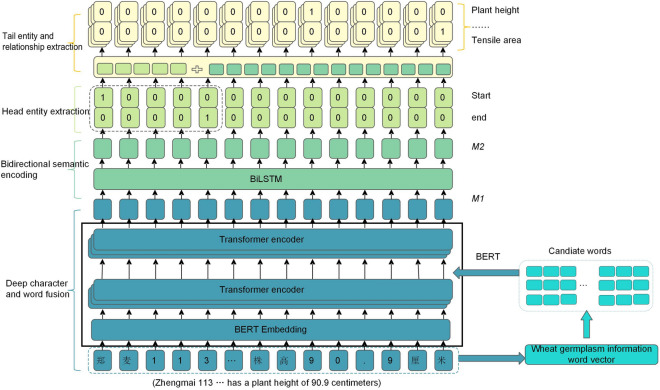
The architecture of the joint extraction of wheat germplasm information entity relationship based on deep character and word fusion(WGIE-DWCF), consists of four parts: deep character and word fusion(DCWFE), contextual semantic information fusion, head entity extraction, and joint extraction of tail entities and relations.

The joint extraction of wheat germplasm information entity relationship based on deep character and word fusion(WGIE-DWCF) model comprised two main layers: the encoding layer and the triple extraction layer. The encoding layer consisted of two components: deep character and word fusion and bidirectional semantic encoding. The triple extraction layer consisted of two components: head entity extraction and joint extraction of tail entities and relations. The overall architecture of the model is illustrated in Fig. 3.

### The encoding layer

#### The deep character and word fusion

**Figure 4:**
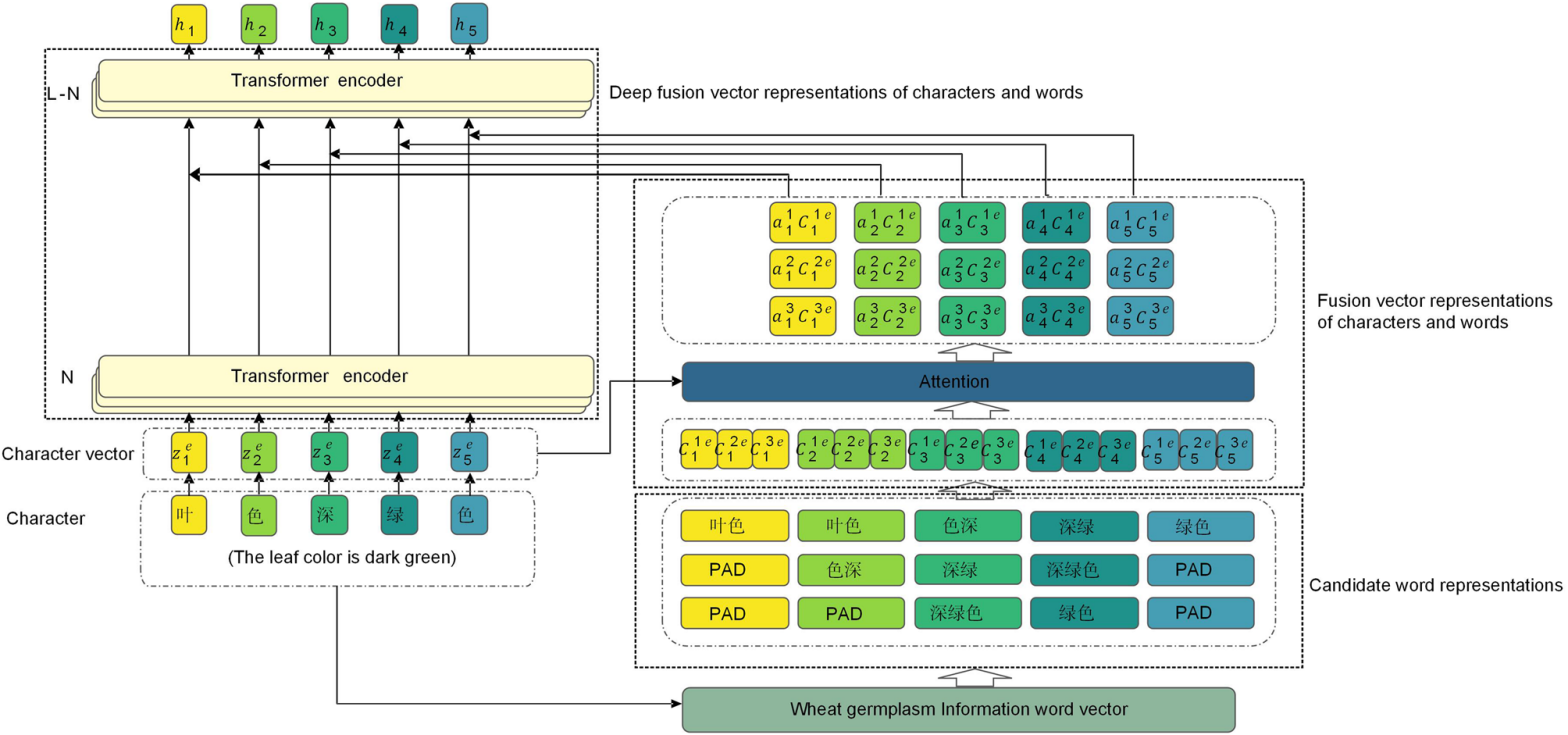
The architecture of the deep character and word fusion encoder (DCWFE) of four components: the wheat germplasm information word vector table, candidate word representation, fusion vector representations of characters and words, and deep fusion vector representations of characters and words.

The deep character and word fusion encoder (DCWFE) integrated word information into the BERT model, alleviating the problem of low entity recognition performance caused by fuzzy boundaries. For each character ${z_i}$ in the input sentence $\{ {z_1},{z_2},...,{z_n}\}$, first, obtain the candidate word vector corresponding to each character. Then, through multiple Transformer encoder layers of BERT, deeply integrate the character and word vectors, thus obtaining deep fusion information of characters and words. The DCWFE module consists of several key components, including the wheat germplasm word vector table, candidate word representations, fusion vector representations of characters and words, and deep fusion vector representations of characters and words. These components worked collaboratively to incorporate word information into the BERT. The structure of the DCWFE module is depicted in Fig. 4.

- The wheat germplasm information word vector table. In order to achieve a unified knowledge representation of wheat germplasm information, this paper established a wheat germplasm information word vector table based on both general domain word data and wheat germplasm domain word data. The general domain word was obtained from Tencent Word Vectors^28^, while the wheat germplasm domain word was trained using fastText^29^ to compute word embeddings. As a result, the wheat germplasm information word vector table was created, containing 20,300 vectors with a dimensionality of 200.
- Candidate word representations. First, for each character ${z_i}$ in the input sentence s=$\{ {z_1},{z_2},...,{z_n}\}$ all candidate words ${c_i}$ matching ${z_i}$ are searched in the wheat germplasm information word vector table. Then, each character ${z_i}$ is paired with all candidate words ${c_i}$ matching it to form character-word pairs. For example, for the character "deep", the character-word pairs matched by the wheat germplasm information word vectors would be (deep, [dark, dark green, dark green color]). Finally, the character ${z_i}$ and its corresponding word ${c_i}$ are converted into corresponding vector representations ${z_i^e}$ and ${c_i^e}$.
- Fusion vector representations of characters and words. When fusing the character and word vectors, an attention mechanism was employed to assign weights to the candidate words, taking into consideration their varying importance. First, a value *m* was set to determine the number of words matched . If the number of candidate words is lower than the *m*, ”PAD” was used for padding. Subsequently, by utilizing the bilinear weight matrix ${W_{atnn}}$, the attention weights $a_i$ between the character vector ${z_i^e}$ and all its candidate word vectors ${c_i^e}$ are computed. Next, based on the attention weights ${a_i^j}$ of the character ${z_i^e}$ corresponding to the word ${c_i^{je}}$, a weighted sum of all candidate word vectors for that character is obtained, thus yielding the final word vector representation $\widetilde{c}$ for the character ${z_i^e}$. Finally, concatenating the character vector ${z_i^e}$ with the final word vector $\widetilde{c}_i$ results in a shallow fusion vector representation of characters and words $\widetilde{h}_i$, as depicted in Eqs. (1) to (3).
  1 $$\begin{aligned} {a_i}= softmax(z_i^e{W_{atnn}}{c{_i^e}^T}) \end{aligned}$$
  2 $$\begin{aligned} \widetilde{{c_i}} = \sum \limits _\mathrm{{j} = 1}^\mathrm{{m}} {a_\mathrm{{i}}^j} c_i^{je} \end{aligned}$$
  3 $$\begin{aligned} \widetilde{{h_i}} = z_i^e + \widetilde{{c_i}} \end{aligned}$$
- Deep fusion vector representations of characters and words. Let $\widetilde{h}_i$ be the vector representation obtained after character and word fusion in the N Transformer encoder of BERT. It served as the input for the N+1 Transformer encoder and then underwent encoding by (L-N) Transformer encoders, resulting in the deep character and word fusion vector representation M1. Here, L represented the total number of Transformer encoders in BERT. This process fully utilized the layer structure of BERT’s Transformers, enabling deep interaction and fusion of both character and word semantic information. As a result, more comprehensive semantic features were obtained. The detailed steps of deep character and word fusion were illustrated in Algorithm 1.

**Algorithm 1:**
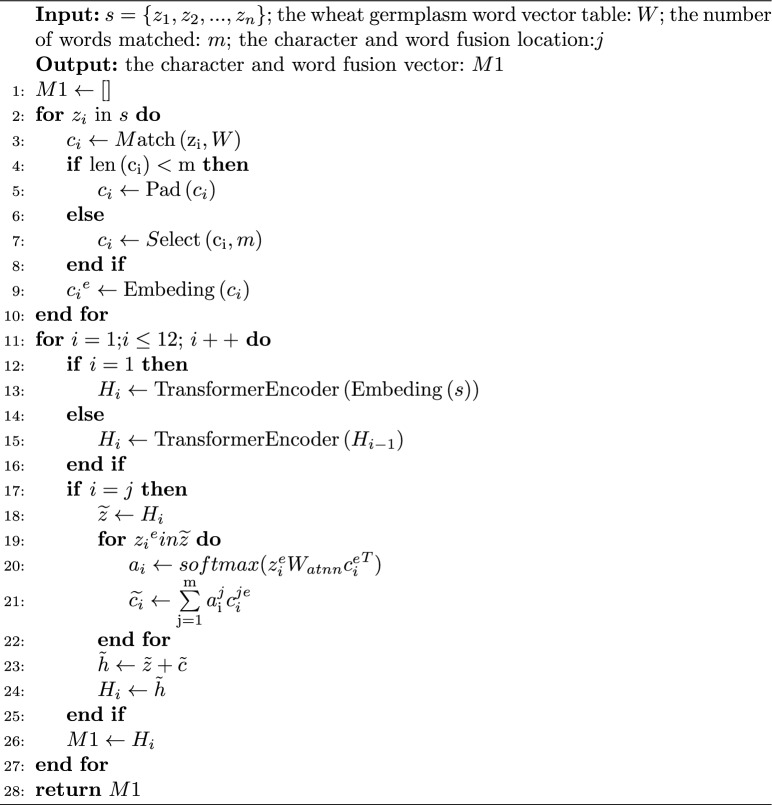
DCWFE Algorithm

#### Bidirectional semantic encoding

In the task of the joint entity and relation extraction, both forward and backward information in the text were crucial. For example, in the entity "Zhongmai 159", the character "mai" should not only capture the forward information of the character "Zhong" but also consider the semantic information of the backward information "159". To address this issue, BiLSTM was chosen to further extract features from the deep character and word fusion vector. The deep fusion vector M1 was used as input, and the concatenated forward and backward information vector M2 was obtained as the output.

### The triple extraction layer

#### Head entity extraction

The purpose of head entity extraction was to identify all possible entities in the input text. It directly decoded the vector M2, which had undergone a deep fusion of characters and words and bidirectional semantic encoding, to determine the start and end positions of the entities. The process was described by equations (4) and (5) as follows:

4 $$\begin{aligned} p_i^{star{t_s}} = Sigmoid({W_{star{t_s}}}M{2_i} + {b_{star{t_s}}}) \end{aligned}$$

5 $$\begin{aligned} p_i^{en{d_s}} = Sigmoid({W_{en{d_s}}}M{2_i} + {b_{en{d_s}}}) \end{aligned}$$

The probabilities $p_i^{star{t_s}}$ and $p_i^{en{d_s}}$ represented the likelihood of the ith token in the input text being the start and end of a head entity, respectively. If the probability exceeded a threshold, the corresponding position in the start or end array was labeled as "1". Otherwise, it was labeled as "0".

**Figure 5:**
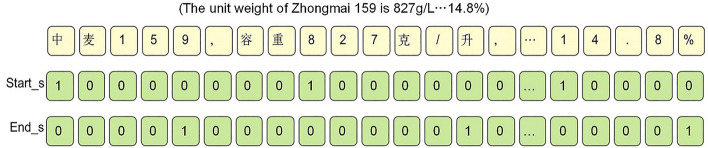
The header entity extraction process. Annotate the start and end positions of all possible entities in the input sentence.

The extraction of candidate head entities followed the "principle of proximity". In Fig. 5, the start positions of the candidate entities in the input text were "Zhong", "8", and "1", while the end positions were "9", "L", and "%". In this case, "8" was selected as the start position of the head entity. According to the "principle of proximity", the closest end position to "8" that appears after it was "L". Therefore, the candidate entity was "827g/L".

The problem of ambiguous entity boundaries can be effectively addressed through deep character and word fusion and bidirectional semantic information encoding. In Fig. 5, an entity "827g/L" is presented. Since this entity consists of both numerical values and units, its boundaries may not be clearly defined, potentially leading to mystification as "827g". However, by incorporating the word information "g/L" and leveraging BERT encoding for the deep fusion of character and word information and bidirectional semantic information encoding, accurate identification of the entity "827g/L" can be achieved.

#### Joint extraction of tail entities and relations

The task of relation and tail entity extraction was to find all candidate tail entities for each specific relation given the candidate head entities. For example, in the text "Zhongmai 159, 827 g/L unit weight, crude protein content (dry basis) 14.8%", after performing the task of head entity extraction, we obtained three candidate head entities: "Zhongmai 159", "827 g/L", and "14.8%". We then proceed to establish 23 types of relations defined in Section "The Annotation Strategy for Wheat Germplasm Information Data", such as "wet gluten content", "unit weight", and "crude protein content" for each of these candidate head entities. Finally, we detected the candidate tail entities for each relation. The process is described in Eqs. (6) and (7).

6 $$\begin{aligned} p_i^{star{t_o}} = Sigmoid({W_{star{t_o}}}(M{2_i} + v_k^{sub}) + {b_{star{t_o}}}) \end{aligned}$$

7 $$\begin{aligned} p_i^{en{d_o}} = Sigmoid({W_{en{d_o}}}(M{2_i} + v_k^{sub}) + {b_{en{d_o}}}) \end{aligned}$$

The vector $v_k^{sub}$ represents the k-th candidate head entity obtained from the head entity extraction task. It is added to the vector M2i, which was the vector representation of the ith token in the encoded text vector M2 obtained from the encoding layer. This combined vector was then decoded to determine the probabilities of the ith token being the start and end positions of the tail entity for a specific relation category. Finally, the corresponding tail entity for the head entity was obtained based on these probabilities.

## Experiment

### Data set and evaluation indicators

This paper conducted experiments on two datasets: WGD and DuIE. The DuIE^30^ dataset, publicly available, is a large-scale information extraction dataset that has been manually annotated by Baidu. It is widely utilized in research endeavors within the public domain. The training set, validation set, test set, and the number of relations for both datasets are shown in Table 1.

**Table 1:** Dataset division statistics table.

Dataset	Relation	Train	Dev	Test
DuIE	17	555956	11191	13417
WGD	23	9344	1169	1168

The effectiveness of the model was verified by calculating the triple extraction results through Precision (P), Recall (R), and F1 values. The evaluation metrics were calculated as shown in Eqs. (8) to (10). $N_{pred}$, ${N_{pred}}^{right}$, $N_{gold}$ were the number of predicted triples, the number of correctly predicted triples, and the number of triples contained in the dataset, respectively.

8 $$\begin{aligned} Precision = \frac{{{N_{pred}}^{right}}}{{{N_{pred}}}} \end{aligned}$$

9 $$\begin{aligned} Recall = \frac{{{N_{pred}}^{right}}}{{{N_{gold}}}} \end{aligned}$$

10 $$\begin{aligned} {F_1} = \frac{{2 \times Precision \times Recall}}{{Precision + Recall}} \end{aligned}$$

### Experimental environment and parameter settings

The hardware environment for this experiment: the processor is Intel(R) Xeon(R) Silver4116 CPU@2.10GHz, running memory 191GB; running environment: Pytroch 1.10.0 and Python 3.6. Using Adam optimizer. The model parameters are set as shown in Table 2.We set the number of words matched per character to 3, the word embedding dimension to 200, the maximum sentence lengths for the WGD and DUIE datasets to 256 and 200 respectively, the BiLSTM dimension to 768, and the learning rate and batch size to 1e-5 and 4 respectively.

**Table 2:** Model parameter values.

Parameter	Value
Number of words matched	3
Word embedding size	200
WGD max sentence length	256
DUIE max sentence length	200
BiLSTM dim	768
Learning rate	1e-5
Batch size	4

### Experimental results and analysis

#### Analysis of deep character and word fusion position settings

**Figure 6:**
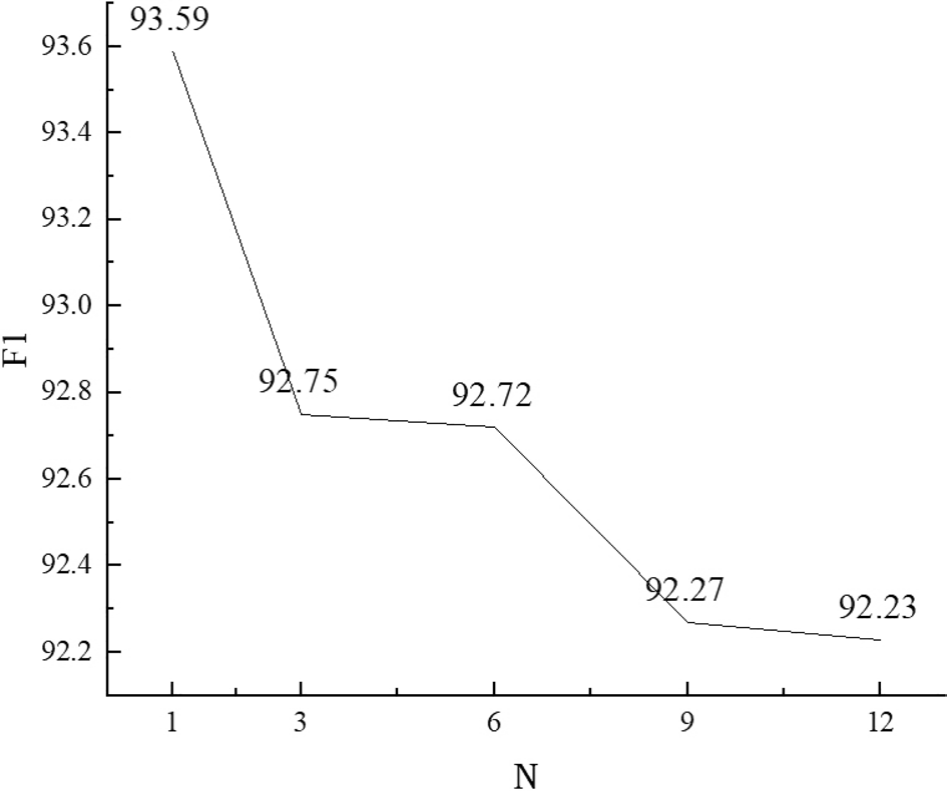
The depth analysis of character and word fusion.

To validate the impact of the position of deep character and word fusion, experiments were conducted by setting the position after the Nth Transformer encoder in BERT, where N = {1, 3, 6, 9, 12}. The trend of F1 score variations is shown in Fig. 6.

When the position of deep character and word fusion was set after the 1st Transformer encoder, the model achieved the optimal performance with an F1 score of 93.59%. As the fusion position was moved further back, the model’s performance gradually decreased. When the fusion was performed after the 12th Transformer encoder, representing shallow character and word fusion, the F1 score was the lowest at 92.23%. This indicated that fusing the character and word vectors after the first encoder layer, effectively leveraging the BERT structure, allowed for better deep interaction between word and character information. As a result, it achieved a deep fusion of semantic information from both characters and words, enhancing the semantic representation capability.

#### Analysis of the parameter setting for the number of character matching words

**Figure 7:**
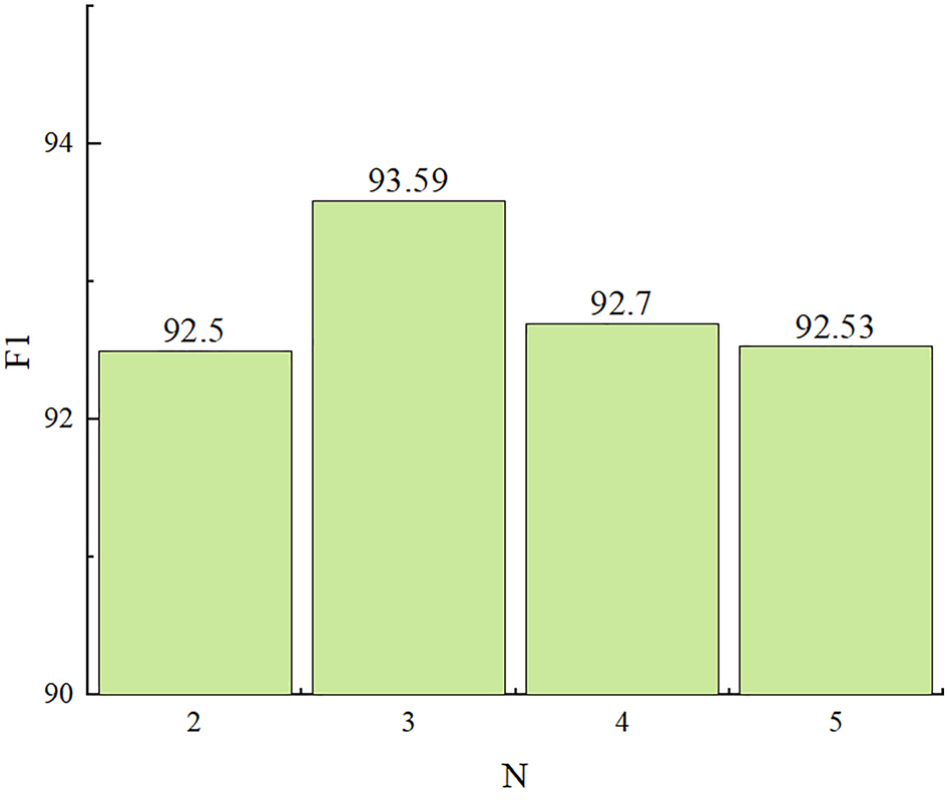
Result in different numbers of matched words.

According to the statistics, the WGD dataset had a mode of 2 for the number of word matches, an average of 3, and a maximum of 5. Therefore, in the experiment, the optimum for the number of word matches was set as N, where N = {2, 3, 4, 5}. The F1 scores corresponding to these optimum values are shown in Fig. 7.

When the optimum for the number of word matches per character was set to 3, the model achieved the best performance. Increasing the value from 2 to 3 allowed the model to access more word vector information, thereby improving its performance. However, as the number of word matches per character continues to increase, the introduction of "PAD" padding information could interfere with the model’s ability to extract features, leading to a gradual decrease in the F1 score. Therefore, the parameter value of the number of words match is set to 3.

#### Analysis of comparative experimental results

To evaluate the effectiveness of the WGIE-DCWF method, this paper compared it with the BERT+BiLSTM, TPLinker^31^, CopyMTL^32^, and BERT+CASREL^8^ models on the WGD and DuIE datasets.

1. BERT+BiLSTM: This model is a pipelined extraction approach that utilizes BERT and BiLSTM for encoding. It performs entity recognition and then identifies the relationship categories between the recognized entities.
2. TPLinker: This model utilizes a joint extraction method based on a unified tagging framework, using BERT word embedding representations.
3. CopyMTL: This model is an improvement based on CopyRE^13^. It used BiLSTM for encoding and incorporates the copy mechanism and multi-task learning for joint extraction.
4. BERT+CASREL: This model is built upon BERT word embeddings and employs a cascading pointer network for joint extraction of entity relationships in triplets.

The experimental results of the models are presented in Table 3. The proposed WGIE-DCWF model achieved the highest F1 scores of 93.59% on the WGD dataset and 77.73% on the DuIE dataset, outperforming other baseline models. Additionally, the joint entity and relation extraction models ((TPLinker, CopyMTL, BERT+CASREL, WGIE-DCWF ) outperformed the pipeline extraction model (BERT+BiLSTM) overall. This was mainly due to the potential entity redundancy and error propagation in the pipeline method. Furthermore, the BERT+CASREL model demonstrated higher precision, recall, and F1 score than the CopyMTL model and TPLinker model, indicating its superior performance among the joint extraction models. The reason was that TPLinker encounters matrix sparsity issues when the sentence length is long, and CopyMTL did not utilize pre-trained language models. Therefore, in this study, the BERT+CASREL model was utilized for a deep fusion of word and character vectors and contextual semantic feature fusion at the encoding layer. This effectively enhanced the entity and relation extraction capability of the WGIE-DCWF model by leveraging both word information and contextual semantic information. In the experiments conducted on the DuIE dataset, the WGIE-DCWF model exhibited lower recall than the BERT+CASREL model. This could be attributed to the introduction of redundant information from the wheat germplasm word vectors in the deep fusion encoding, which affected the model’s joint extraction performance on a general dataset.

**Table 3:** Model performance evaluation.

Model	WGD			DuIE		
	P/%	R/%	F1/%	P/%	R/%	F1/%
BERT+BiLSTM	52.80	47.30	49.76	46.7	32.2	38.1
TPLinker	61.28	60.00	60.63	62.41	73.43	67.47
CopyMTL	63.88	60.21	61.99	49.9	39.4	43.9
BERT+CASREL	74.77	92.91	82.86	70.67	76.15	73.31
WGIE-DCWF	92.96	94.24	93.59	81.14	74.59	77.73

#### Analysis of the results of the fine-grained relation experiments

To analyze the experimental results of the WGIE-DCWF model at different levels of relationship granularity, the precision, recall, and F1 scores for the 23 relationship categories on the WGD dataset were calculated and are presented in Table 4.

**Table 4:** Experimental results of fine-grained relation extraction.

Relation	P/%	R/%	F1/%	Relation	P/%	R/%	F1/%
Growth period	95.69	93.49	94.57	Winter-spring type	95.44	94.36	94.90
Plant height	93.52	95.47	94.48	Maturity	85.56	95.06	90.06
Spike number per mu	94.05	94.42	94.23	Shell color	95.98	95.56	95.77
Kernel number	96.22	95.24	95.73	Seed color	92.61	94.95	93.76
1000-grain weight	93.63	93.93	93.78	Grain hardness	93.63	93.63	93.63
Unit weight	94.59	94.59	94.59	Grain shape	92.31	89.55	90.91
Stability time	96.12	95.59	95.86	Awn length	95.07	96.43	95.74
Tensile area	96.61	97.44	97.02	Leaf color	95.07	94.15	94.61
Yield	95.13	95.74	95.43	Plant type	92.31	95.77	94.01
Sowing time	90.10	90.72	90.41	Crude protein content	93.95	96.75	95.33
Seeding quantity	85.39	83.21	84.29	Wet gluten content	93.63	95.92	94.77
Disease susceptibility	87.57	93.09	90.25				

The "stability time" and "crude protein content" relationship categories had higher F1 scores, which might be due to an adequate number of samples for these categories, allowing the model to learn their features effectively. On the other hand, the "grain shape" and "maturity" relationship categories, which accounted for only 0.87% and 1.14% of the samples, respectively, had lower F1 scores compared to other categories. This suggested that the limited number of samples for these categories could be easily overlooked during model training, resulting in lower extraction performance compared to categories with higher sample counts. The relationship categories of "tensile area", "awn length", "winter-spring type" and "shell color" showed good extraction performance, possibly due to their simpler contextual information. On the other hand, the relationship categories of "seeding quantity", "sowing time", "disease susceptibility ", and "grain hardness" had lower F1 scores than the average. This could be attributed to the long-distance dependency between the head entity and the tail entity, as well as the complexity of the context. For example, the relationship category of "seeding quantity" could be associated with entities such as wheat names, seeding quantity under high-fertilizer and high-water conditions, and basic seedling numbers. Hence, the distribution of samples and the complexity of the context significantly affected the performance of entity relation extraction.

#### Analysis of the results of the ablation experiment

To explore the impact of deep character and word fusion(DCWFE), bidirectional semantic encoding(BiLSTM), and the wheat germplasm information word vector table on the WGIE-DCWF method, ablation experiments were designed. The experimental results are shown in Table 5. In this table, DCWFE* represented the deep word and character fusion with the wheat germplasm word vector table containing only general vocabulary, excluding domain-specific vocabulary.

**Table 5:** Results of ablation experiments.

Model	P/%	R/%	F1/%
BERT+CASREL	74.77	92.91	82.86
BERT+ BiLSTM +CASREL	92.56	92.77	92.66
DCWFE +CASREL	92.38	93.53	92.95
DCWFE* + BiLSTM +CASREL	92.47	93.23	92.85
DCWFE+ BiLSTM +CASREL	92.96	94.24	93.59

The analysis of the experimental results revealed that the introduction of deep character and word fusion and bidirectional semantic encoding led to an increase in the F1 score by 10.09 percentage points and 9.8 percentage points, respectively. This indicated that both parts individually contribute to improving the overall performance of the model. Furthermore, it highlighted the equal importance of deep character and word and bidirectional semantic encoding, with deep character and word fusion having a slightly greater impact on the model’s performance. To understand the role of domain-specific word vectors in the model, it could be observed that the DCWFE* + BiLSTM + CASREL model showed a decrease of 0.74 percentage points in the F1 score. This suggested that the introduction of domain-specific word vectors could enhance entity recognition ability, thereby assisting in triple extraction.

## Conclusion

In order to obtain structured phenotypic and genetic descriptions of wheat varieties, this study constructed a fine-grained wheat germplasm dataset, addressing the scarcity of data in the wheat germplasm domain. It proposed the WGIE-DCWF model. The model enhances entity recognition by integrating deep character-word fusion with contextual semantic features and establishes a cascading pointer network to improve the extraction of overlapping triples. Experimental results demonstrate that the proposed model outperforms other models, mitigating the impacts of entity boundary ambiguity and relationship overlap, effectively improving the extraction of entity relationships in complex scenarios within the wheat germplasm domain. This model can support the construction of knowledge graphs and intelligent question-answering systems in the wheat germplasm domain, which is important for crop breeding, genetic research, cultivation management, and agricultural production.

In future work, we will introduce relation type embedding representations to alleviate the issue of low performance in identifying relation categories with few samples, thereby improving the overall performance of the entity relation joint extraction model. In addition, we will conduct further research such as diversity analysis of varieties to explore deeper associations within the wheat germplasm information data. This will help researchers and breeders better understand the genetic background of wheat and select high-quality varieties more effectively.

## Data Availability

The datasets used or analyzed during the current study available from the corresponding author on reasonable request.
